# Psychotic experiences and psychological distress in adolescents: an examination of longitudinal bidirectional effects across sex

**DOI:** 10.1186/s13034-024-00825-w

**Published:** 2024-10-03

**Authors:** Feten Fekih-Romdhane, Lilia Houissa, Alexandre Andrade Loch, Majda Cheour, Souheil Hallit

**Affiliations:** 1 The Tunisian Center of Early Intervention in Psychosis, Department of Psychiatry “Ibn Omrane”, Razi hospital, 2010 Manouba, Tunisia; 2 Faculty of Medicine of Tunis, Tunis El Manar University, Tunis, Tunisia; 3 Laboratorio de Neurociencias (LIM 27), Instituto de Psiquiatria, Hospital das Clinicas HCFMUSP, Faculdade de Medicina, Universidade de Sao Paulo, Sao Paulo, SP Brazil; 4 Instituto Nacional de Biomarcadores em Neuropsiquiatria (INBION), Conselho Nacional de Desenvolvimento Cientifico e Tecnológico, Sao Paulo, Brazil; 5 School of Medicine and Medical Sciences, Holy Spirit University of Kaslik, P.O. Box 446, Jounieh, Lebanon; 6 Psychology Department, College of Humanities, Effat University, 21478 Jeddah, Saudi Arabia; 7 Applied Science Research Center, Applied Science Private University, Amman, Jordan

**Keywords:** Psychotic experiences, Adolescents, Cross-lagged panel models, Sex moderation

## Abstract

**Background:**

Although the co-occurrence of psychotic experiences (PEs) and psychological distress symptoms is growingly recognized in several previous studies, there is still a lack of literature, which clearly outlines how these two psychopathological entities affect each other over time. This study is intended to add to the literature by examining: (a) the longitudinal, bidirectional associations between PEs and psychological distress in a sample of Tunisian adolescents, and (b) whether these associations are moderated by sex.

**Methods:**

510 adolescent students (mean age of 16.05 ± 1.01 years, 61.2% females) took part in a prospective longitudinal study. PEs and psychological distress were measured at three occasions over a one-year period. The cross-lagged panel modeling approach was adopted.

**Results:**

The current results showed that the experience of PEs at baseline tended to temporally precede prospective increases in psychological distress (at 6 months), which had in turn led to further exacerbation of psychological distress at 12 months of follow-up. Temporally primary psychological distress symptoms were not a significant predictor for the development of later psychotic symptoms in the whole sample. However, temporal patterns between adolescent distress and psychotic symptoms differed for girls and boys. Endorsing PEs at baseline was followed by greater psychological distress at 6 months, which was in turn associated with a significant increased risk of subsequent exacerbation of PEs at 12 months in boys, whereas psychological distress at 12-month follow-up was significantly predicted by pre-existing PEs in girls.

**Conclusion:**

These findings suggest that clinicians and support workers are recommended to take into account different social risk profiles for boys and girls when considering interventions to address PEs and distress in adolescents.

## Introduction

Psychotic experiences (PEs) encompass beliefs, thoughts, or perceptions that are considered unreal, unusual or odd (i.e. sub-threshold hallucinations and delusions) occurring with less frequency and milder severity, in the absence of a psychotic disorder [1, 2]. They are positioned at the lower end of the extended psychosis continuum, and are frequently experienced by people from the general non-help-seeking population [2]. Prevalence estimates of PEs were reported to be 7.5% [3], varying substantially across cohorts and studies [4, 5]. They are most prevalent among children and adolescents [3]. Although some individuals who experience PEs are unlikely to ever convert to psychosis in their life, PEs were found to be significant predictors and risk markers for the development of later psychotic disorders [1, 6], and have therefore attracted considerable research interest in terms of their potential in detecting and predicting psychosis risk status in the general population [7]. In addition, PEs may denote young people’s vulnerability to broader psychopathology, and qualifies as a transdiagnostic marker for a range of mental disorders [8].

Indeed, young people who endorse transitory PEs showed an increased likelihood to express both externalizing and internalizing symptoms [9–11], and those who experience persistent PEs were found to be more likely to suffer from adverse mental health outcomes and to perceive a need for care [1, 12]. According to a meta-analysis, the occurrence of PEs is associated with greater than twofold increase in the likelihood of reporting mental health service use [13]. The possible negative outcomes of PEs include chronic physical [14] and mental [15] health conditions, behavioral and addictive problems [16–20], suicidality [21, 22], reduced lifespan [23], disability [24], as well as psychological distress [25]. Therefore, it appears that PEs can potentially play a pivotal role in early detection, prevention, and intervention for a broad array of psychopathology, provided that pathways linking PEs and psychopathology are clearly understood. The present research proposes to shed light on these pathways by exploring the reciprocal relationships between PEs and psychological distress.

### Bidirectional associations between PEs and psychological distress

Psychological distress is generally expressed as a state of emotional suffering characterized by negative emotions such as anxiety (e.g., feeling tense, restlessness), depression (e.g., sadness, hopelessness, lost interest) and stress [26]. A compelling and growing body of literature indicated that PEs can induce psychological distress, and conversely, psychological distress can contribute to the development or worsening of PEs. For instance, a meta-analysis by Healy et al. [27] revealed that children and adolescents who report PEs have a 3-fold greater risk of developing any mental disorder. A 10-year longitudinal study demonstrated that young people who reported PE are at a significantly increased risk for both a single lifetime mental disorder and multi-morbid disorders, with the most commonly diagnosis met being lifetime major depression followed by anxiety disorders [28]. The results of the Australian Young Minds Matter Survey showed that hallucinatory and delusional experiences endorsed by adolescents aged 14- to 17-year-olds were significantly related to an elevated likelihood of psychological distress [29]. Findings from World Health Organization World Mental Health (WMH) Surveys showed that temporally primary PEs were linked to the subsequent development of a range of mental health problems, including depression, anxiety and post-traumatic stress, and that, in contrast, numerous mental health problems were significant predictors of first-onset of PEs [30]. In contrast, Stochl et al. [31] argued against the concept of co-morbidity between psychotic phenomena and depression/anxiety symptoms in the general population. They rather suggest that, at a subclinical level, these manifestations reflect the same psychopathological concept and represent a unitary underlying continuum of “common mental distress”, with PEs being at the upper end of severity [31].

At the same time, some controversial findings have been reported. For instance, Wigman et al. [32] evaluated 138 adolescent help-seekers treated for depression at 4 time-points over a two-year period, and found that PEs and depressive symptoms did not predict each other longitudinally. Nonetheless, the statistical power of these findings might have been limited by sample size [32]. To sum up, there is longitudinal evidence in diverse clinical and non-clinical populations showing that PEs are related to subsequent depression (e.g [33–37]), and that depression is related to later psychotic symptoms (e.g [38, 39]). These previous findings suggest that PEs and psychological distress are interrelated, and that arrows of causality could possibly run in both directions. However, this topic and field of study has paid relatively little attention to understanding temporal patterns of co-occurrence between PEs and psychological distress in non-clinical samples with milder forms of distress, which is determinant for evidence-based prevention and intervention practices. At last, previous evidence suggests that sex is an important consideration when examining the relationship between PEs and distress.

### Moderating effect of sex

There are multiple plausible theoretical and empirical reasons why sex moderation is expected in this study. The sex disparity in rates of psychological distress, such as depression where girls predominate in a 1.95 ratio over boys, emerges during the adolescent years and persists into adulthood [40, 41]. The observed sex differences in psychological distress are due to the joint influence of biological, cognitive, affective, and sociocultural determinants [41]. Prior research has also pointed to differences in how males and females express and experience their psychotic symptoms that extend across the psychosis continuum [42]. Females tend to endorse more PEs [43]; however, opposite findings have also been reported [44, 45]. In addition, sex differences were noticed regarding aspects of psychotic symptoms, with female adolescents being more likely to experience persecutory ideation and subclinical hallucinations compared to males [46, 47]. Altogether, these observations advocate that sex differences in interrelationships between PEs and psychological distress are plausible. However, there is only a paucity of studies specifically focusing on how the associations between PEs and distress may differ across sex, and this research question is yet to be addressed. To fill this gap in the literature, the present study included sex as a moderating variable.

### Rationale and purpose of the present study

Adolescence represents the peak age of emergence of mental illness [48], as well of PEs, and is therefore a critical period for inquiry into factors that pose a risk to mental health. The reciprocal time-lagged associations between PEs and psychological distress are still poorly understood and under-researched, as most prior research has examined unidirectional effects. Elucidating these associations in a community sample of adolescents offers an opportunity to gauge the extent to which the occurrence of PEs leads to psychological distress, and whether temporally primary distress predicts the subsequent first onset of PEs. This might provide significant clinical implications for prevention and early intervention, as research showed that the intersection between PEs and nonpsychotic symptoms/disorders confers substantial public mental health burden. Indeed, the co-occurrence of the two entities was found to be associated with great functional deficits (e.g [49]), higher proportions of lifetime suicide attempt reporting, more use of psychological treatment, and more elevated odds of mental health services use [50]. Furthermore, large cross-country and cross-cultural differences have been seen in the way distress [51], PEs [52], and sex gaps in mental health [53] are expressed. Although cross-sectional associations between hallucinatory/delusional symptoms and increased anxiety, depression and stress have been documented in samples from Arab countries [18, 54–58], the directionality and nature of these associations remain to be disentangled, and particularly in the context of a longitudinal design. This study is intended to add to the literature by examining: (a) the longitudinal, bidirectional associations between PEs and psychological distress using cross-lagged panel modeling approach in a sample of Tunisian high-school adolescent students, and (b) whether these associations are moderated by sex. Guided by previous research, it is expected that there will be a bidirectional association between PEs and distress symptoms, such that PEs will act as contributor to distress, and that distress will be a significant predictor of later PEs. In addition, it is anticipated that positive associations between PEs and distress will be stronger in both directions for girls as compared to boys.

## Methods

### Sample and procedure

A prospective longitudinal study was performed over a 12-month period (April 2022-April 2023), by adopting a non-probability convenience sampling technique. The target population was high-school students from four high schools in Tunis, Tunisia. Students were approached in their classrooms after lectures. They were eligible to participate if they: (1) were aged 13–18 years, (2) had no personal history of psychosis or antipsychotic medication intake, (3) had a French language proficiency, and (4) have completed the three scheduled assessments. Of the 1692 students initially approached to participate, 510 were included in the final analyses (Further details on the study sample are provided in [59]).

### Ethics

The study was performed following the Declaration of Helsinki for human research. Ethical approval was provided by the ethics committee of the Razi psychiatric hospital, Manouba, Tunisia. Before approaching students, approval was obtained from school authorities and the parent or legal guardian after a first contact held to introduce the research project. In addition, each participating student provided a voluntary oral informed assent before beginning the survey. All participants and their parent(s)/legal guardian(s) were informed that the study necessitates a three-point assessment procedure.

### Measures

Participants took part of three-time-point assessments: at baseline (T0), 6 months (T1) and 12 months (T2). They were invited to complete a self-report paper-and-pencil questionnaire in the French language composed of two sections. The language of the questionnaire was not considered as a barrier to participants’ completion of the survey, as it represents the language of high-school studies in the country. The first section contained demographic information, while the second section contained two scales: The “Community Assessment of Psychic Experiences” (CAPE-42) and the Depression, Anxiety, Stress Scales-21 (DASS-21). Sociodemographic data collected consisted of age, sex (male, female), and residency (rural, urban). In addition, the socioeconomic status was reflected through the participants’ monthly family income in Tunisia Dinars (i.e. <1000, 1000–2000, 2000–3000, > 3000) as done in previous studies among Tunisian students (e.g [60–62]).

#### The CAPE-42

This is self-administered scale composed of 42 items that assess positive, negative and depressive symptoms through two dimensions (i.e., frequency of symptoms and related distress) [63]. The frequency dimension is scored on a four-point scale from 1 (never) to 4 (nearly always), while the distress dimension is rated from 1 (not distressed) to 4 (very distressed). Only the positive CAPE subscale, which is composed of 20 items, was used. Total scores for the CAPE positive subscale range from 20 to 80 on both dimensions. Higher scores reflect greater PEs. The French version adopted demonstrated excellent psychometric qualities [64], and was previously used in non-clinical Tunisian populations [65–67]. The present sample yielded Cronbach alpha values for the positive CAPE dimension ranging from 0.817 to 0.849 for the three time points.

#### The DASS-21

This is a 4-point Likert-type measure composed of 21 items, which are divided into three subscales of seven items each, reflecting the severity of anxiety, depression and stress symptoms [68]. Scores range from 0 to 21 for each dimension, and from 0 to 63 for the DASS-21 total score. The DASS-21 has showed good internal consistency in different non-clinical and clinical samples [69]. The DASS-21 also showed good psychometric qualities among adolescents across different countries [70]. For the current sample, the reliability of the DASS-21 total score was good (Cronbach’s alpha ranging from 0.897 to 0.917 for the three time points).

### Data analysis

**Fig. 1:**
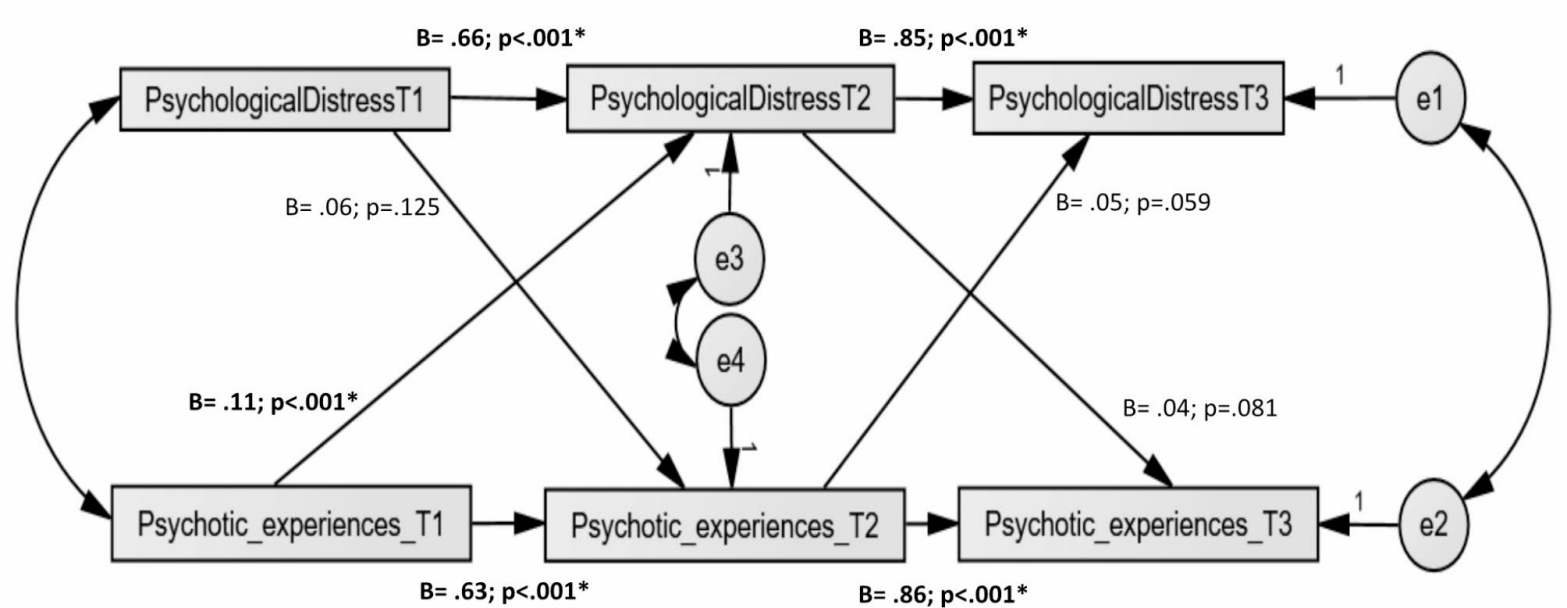
Structural equation model examining the associations between psychological distress and positive dimensions in the total sample. Rectangles indicate observed variables. Small circles reflect residuals (e); Unidirectional arrows depict hypothesized directional associations. Models were adjusted over age and sex but are not presented visually for parsimony. (*Standardized* Beta = 0.06, *p* = 0.125)

We used SPSS AMOS v.28 to examine the bidirectional relationship between psychological distress and CAPE positive dimension over time and to assess model fit. The scores were considered normally distributed as their skewness and kurtosis values varied between + 1.96, -1.96 [71]. We employed the structural equation modeling approach to test the hypothesized model. To address the primary purpose of the study, we tested a cross-lagged model, including both the autoregressive effects to examine the temporal stability of psychological distress and CAPE positive dimension across time, and the regression effects to examine the bidirectional relationship between psychological distress and CAPE positive dimension. The autoregressive effects included a path from psychological distress at Time 1 to Time 2 to Time 3, and a second path from CAPE positive dimension at Time 1 to Time 2 to Time 3. The regression effects included a path from psychological distress at Time 1 to CAPE positive dimension at Time 2 to psychological distress at Time 3, and a path from CAPE positive dimension at Time 1 to psychological distress at Time 2 to CAPE positive dimensions at Time 3. For standardized Beta coefficients of the cross-lagged model, the values of 0.03, 0.07, and 0.12 correspond to small, moderate and high effect sizes respectively [72].The results were adjusted over age and sex; age was a continuous variable, with higher values indicating older age in years; sex was coded as 0 = male and 1 = female. The model also examined synchronous correlations by allowing psychological distress and CAPE positive dimension to intercorrelate within each time point (represented by the curved, double-headed arrows in Fig. 1). We examined the model fit of each of these models and interpreted the significant parameter estimates. To address the secondary purpose of the study, we conducted multiple group analyses to determine whether the relationships between psychological distress and positive dimension differed by sex. We tested the cross-lagged models in each of the prior analyses, while controlling for age. In this set of analyses, the relative fit of an unrestricted model, which allowed parameters to be estimated freely across the two groups (boys versus girls), was compared to the fit of a constrained model that held the paths constant (fixed paths to be the same for both boys and girls). The fit of the models was assessed with the lack of significance (*p* < 0.05) of the chi-square test (χ^2^), the comparative fit index (CFI), the root mean square error of approximation (RMSEA), and the standardized root mean square residual (SRMR). We reported SRMR because of the relatively small sample sizes (*N* < 250). χ^2^df ≤ 5, RMSEA values ≤ 0.08, CFI and TLI values ≥ 0.90, and SRMR values less than 0.05 were used as cut-off points for acceptable model fit [72]. *P* < 0.05 was deemed statistically significant.

## Results

### Descriptive statistics

Five hundred ten participants took part of the study. The mean age of the sample was 16.05 ± 1.01, with 61.2% females. Other characteristics of the sample are shown in Table 1. Table 2 shows the correlation matrix between the scores of positive PEs and psychological distress.

**Table 1:** Sociodemographic and other characteristics of the participants (*n* = 510)

Sex	
Male	198 (38.8%)
Female	312 (61.2%)
Residency	
Rural	11 (2.2%)
Urban	499 (97.8%)
Family income (in Tunisian Dinars)	
< 1000	21 (4.1%)
1000–2000	132 (25.9%)
2000–3000	164 (32.2%)
> 3000	193 (37.8%)

**Table 2:** Correlation matrix

	Mean ± SD	1	2	3	4	5
1. Psychological distress T1	21.32 ± 11.86	1				
2. Psychological distress T2	20.83 ± 12.48	0.75***	1			
3. Psychological distress T3	20.11 ± 11.97	0.71***	0.88***	1		
4. Psychotic experiences T1	39.60 ± 8.74	0.51***	0.47***	0.43***	1	
5. Psychotic experiences T2	39.14 ± 9.07	0.40***	0.53***	0.50***	0.68***	1
6. Psychotic experiences T3	39.71 ± 9.48	0.38***	0.50***	0.52***	0.62***	0.88***

CAPE: Community Assessment of Psychic Experiences

*p < 0.05; **p < 0.01; ***p < 0.001

#### Comparison of variables between sexes

Higher means psychological distress and CAPE positive dimension scores at baseline, 6 and 12 months were significantly found in females compared to males (Table 3).

**Table 3:** Comparison of variables between sexes

	Males (Mean ± SD)	Females (Mean ± SD)	t	df	*p*	95% CI of the difference
Age	16.04 ± 0.96	16.05 ± 1.05	– 0.09	508	0.932	− 0.19; 0.17
Psychological distress T1	16.23 ± 10.44	24.55 ± 11.57	– 8.22	508	**< 0.001**	− 10.32; − 6.34
Psychological distress T2	15.42 ± 10.82	24.25 ± 12.26	– 8.52	508	**< 0.001**	− 10.86; − 6.79
Psychological distress T3	14.57 ± 10.23	23.62 ± 11.67	– 9.22	508	**< 0.001**	− 10.99; − 7.13
Psychotic experiences T1	37.51 ± 8.49	40.92 ± 8.65	– 4.38	508	**< 0.001**	− 4.95; − 1.89
Psychotic experiences T2	36.81 ± 9.06	40.61 ± 8.77	– 4.70	508	**< 0.001**	− 5.38; − 2.21
Psychotic experiences T3	37.31 ± 9.60	41.23 ± 9.10	– 4.64	508	**< 0.001**	− 5.58; − 2.26

Numbers in bold indicate significant *p* values

### Bidirectional associations between psychological distress and PEs (CAPE positive dimension)

Figure 1 presents the results of the autoregressive effects and cross-lagged associations between psychological distress and CAPE positive dimension (PEs), adjusted over age and sex. This model was adequate as shown by the following fit indices: χ^2^/df = 33.08/9 = 3.68, *p <* 0.001; RMSEA = 0.073 [90% CI 0.047, 0.100]; CFI = 0.991; TLI = 0.971 and SRMR = 0.016. Autoregressive paths were significant; Time 1 psychological distress was positively and strongly associated with Time 2 psychological distress, *Standardized Beta* = 0.66, *p* < 0.001, and Time 2 psychological distress with Time 3 psychological distress, *Standardized Beta* = 0.85, *p* < 0.001. In addition, PEs at Time 1 strongly predicted higher PEs at Time 2, *Standardized Beta* = 0.63, *p* < 0.001, which strongly predicted higher PEs at Time 3, *Standardized Beta* = 0.85, *p* < 0.001. Moreover, PEs at Time 1 significantly but weakly increased psychological distress at Time 2 (*Standardized Beta* = 0.11, *p* < 0.001), whereas psychological distress at Time 1 was not significantly associated with PEs at Time 2 (*Standardized* Beta = 0.06, *p* = 0.125). Finally, psychological distress at Time 2 was not significantly associated with PEs at Time 3 (*Standardized Beta* = 0.04, *p* = 0.081), whereas PEs at T2 was not significantly associated with psychological distress at Time 3 (*Standardized Beta* = 0.05, *p* = 0.059).

Moreover, three significant indirect effects were observed in the total sample: (1) psychological distress at Time 2 weakly mediated the association between PEs at Time 1 and psychological distress at Time 3 (Beta = 0.13; 95% CI 0.06, 0.19, *p* = 0.004, (2) PEs at Time T2 strongly mediated the association between positive dimensions at Time 1 and Time 3 (Beta = 0.55, 95% CI 0.46, 0.61, *p* = 0.007, (3) psychological distress at Time 2 strongly mediated the association between psychological distress at Time 1 and Time 3 (Beta = 0.56, 95% CI 0.49, 0.64, *p* = 0.003).

### Moderation analysis by sex

**Fig. 2:**
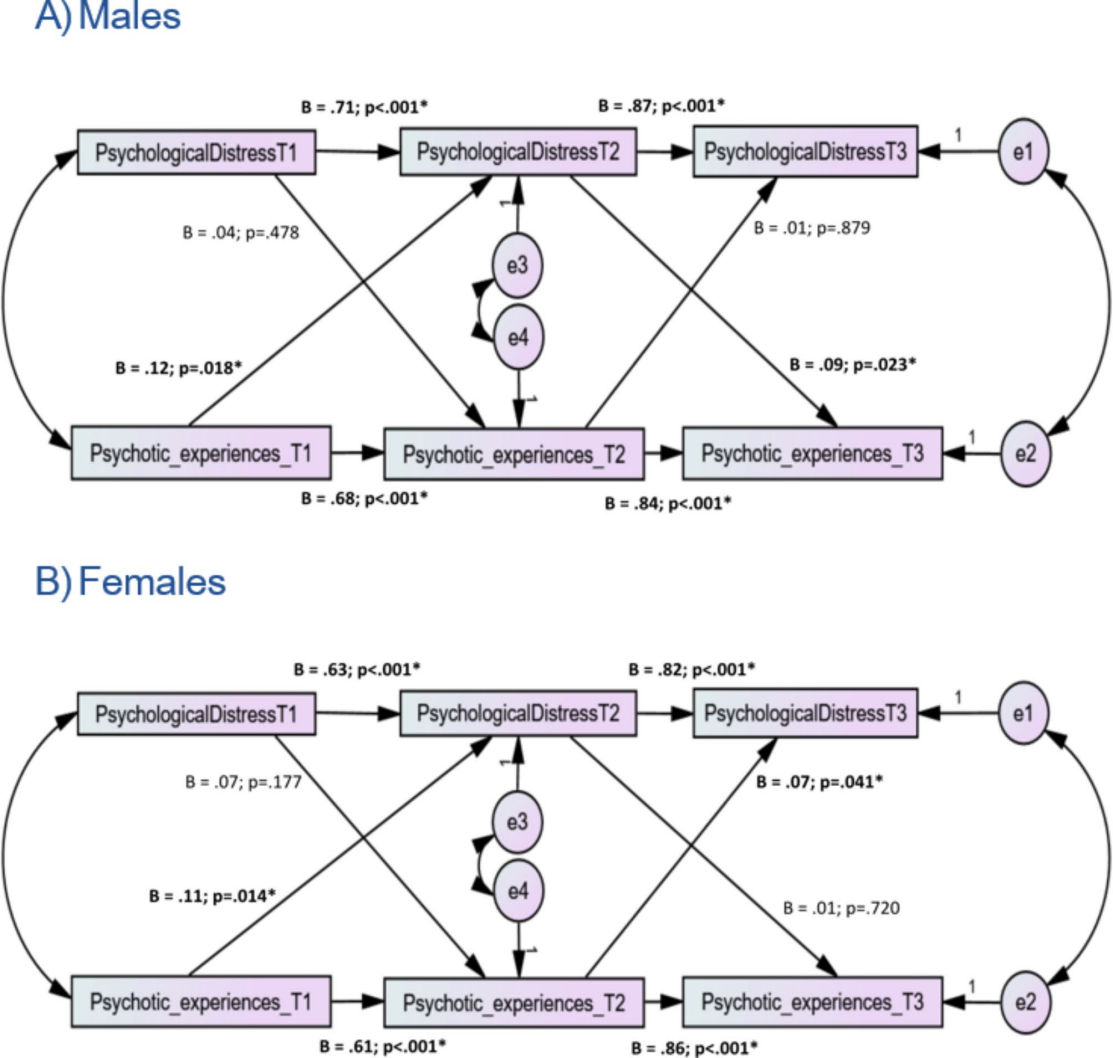
Structural equation model examining the associations between psychological distress and positive dimensions in males (**A**) and females (**B**). Rectangles indicate observed variables. Small circles reflect residuals (e); Unidirectional arrows depict hypothesized directional associations. Models were adjusted over age and sex but are not presented visually for parsimony

Figure 2 presents the results of the autoregressive effects and cross-lagged associations between psychological distress and CAPE positive dimension (PEs), stratified by sex. In the model related to males (Fig. 2A), the fit indices were adequate: χ^2^/df = 16.95/4 = 4.24, *p =* 0.002; RMSEA = 0.128 [90% CI 0.069, 0.194]; CFI = 0.988; TLI = 0.953 and SRMR = 0.019. Autoregressive paths were significant; Time 1 psychological distress was positively and strongly associated with Time 2 psychological distress, *Standardized Beta* = 0.71, *p* < 0.001, and Time 2 psychological distress with Time 3 psychological distress, *Standardized Beta* = 0.87, *p* < 0.001. In addition, PEs at Time 1 strongly predicted higher PEs at Time 2, *Standardized Beta* = 0.68, *p* < 0.001, which strongly predicted higher PEs at Time 3, *Standardized Beta* = 0.84, *p* < 0.001. Moreover, PEs at Time 1 significantly but weakly increased psychological distress at Time 2 (*Standardized Beta* = 0.12, *p* = 0.018), whereas psychological distress at Time 1 was not significantly associated with PEs at Time 2 (*Standardized* Beta = 0.04, *p* = 0.478). Finally, psychological distress at Time 2 was significantly associated with PEs at Time 3 (*Standardized Beta* = 0.09, *p* = 0.023), whereas PEs at T2 was not significantly associated with psychological distress at Time 3 (*Standardized Beta* = 0.01, *p* = 0.879).

The total indirect effect from PEs at T1 and T3 (Standardized Beta = 0.58; 95% CI 0.48, 0.68; *p* = 0.005) was significant, with pathways PEs at T1 → PEs at T2 → PEs at T3 and PEs at T1 → psychological distress at T2 → PEs at T3 being both significant. Furthermore, the total indirect effect from psychological distress at T1 and T3 (Standardized Beta = 0.61; 95% CI 0.50, 0.73; *p* = 0.003) was significant, with pathway psychological distress at T1 → psychological distress at T2 → psychological distress at T3 being significant, while pathway psychological distress at T1 → PEs at T2 → psychological distress at T3 was not significant.

In the model related to females (Fig. 2B), the fit indices were adequate: χ^2^/df = 10.20/4 = 2.55, *p =* 0.037; RMSEA = 0.071 [90% CI 0.016, 0.126]; CFI = 0.995; TLI = 0.982 and SRMR = 0.013. Autoregressive paths were significant; Time 1 psychological distress was positively and strongly associated with Time 2 psychological distress, *Standardized Beta* = 0.63, *p* < 0.001, and Time 2 psychological distress with Time 3 psychological distress, *Standardized Beta* = 0.82, *p* < 0.001. In addition, PEs at Time 1 strongly predicted higher PEs at Time 2, *Standardized Beta* = 0.61, *p* < 0.001, which strongly predicted higher PEs at Time 3, *Standardized Beta* = 0.86, *p* < 0.001. Moreover, PEs at Time 1 significantly but weakly increased psychological distress at Time 2 (*Standardized Beta* = 0.11, *p* = 0.014), whereas psychological distress at Time 1 was not significantly associated with PEs at Time 2 (*Standardized* Beta = 0.07, *p* = 0.177). Finally, psychological distress at Time 2 was not significantly associated with PEs at Time 3 (*Standardized Beta* = 0.01, *p* = 0.720), whereas PEs at T2 was significantly associated with psychological distress at Time 3 (*Standardized Beta* = 0.07, *p* = 0.041).

The total indirect effect from PEs at T1 and T3 (Standardized Beta = 0.52; 95% CI 0.43, 0.61; *p* = 0.004) was significant, with pathways PEs at T1 → PEs at T2 → PEs at T3 being significant while pathway PEs at T1 → psychological distress at T2 → PEs T3 was not significant. Furthermore, the total indirect effect from psychological distress at T1 and T3 (Standardized Beta = 0.52; 95% CI 0.44, 0.62; *p* = 0.003) was significant, with pathway psychological distress at T1 → psychological distress at T2 → psychological distress at T3 being significant, while pathway psychological distress at T1 → PEs at T2 → psychological distress at T3 was not significant. Finally, the total indirect effect of PEs at T1 and psychological distress at T3 (standardized Beta = 0.14; 95% CI 0.05, 0.21; *p* = 0.007) was significant, with pathway PEs at T1 → psychological distress at T2 → psychological distress at T3 being significant, while pathway PEs at T1 → PEs at T2 → psychological distress at T3 was not significant.

## Discussion

Even though the co-occurrence of PEs and psychological distress symptoms is growingly recognized in several major previous studies [33, 34, 73–75], the temporal sequence and causal relationships between these two psychopathological entities cannot be determined on the basis of the current state of the literature. This underlines the need for longitudinal studies investigating both directions between PEs and psychological distress, which may allow to improve understanding of the challenges confronted by adolescents with both conditions and intervene more effectively to promote their mental health. Our findings revealed that the model predicting general symptoms of psychological distress at 6 months after temporally primary PEs was significant, whereas no statistical significance was found for the reverse path of causality in the total sample. These findings, however, were moderated by sex, where endorsing PEs at baseline was followed by greater psychological distress at 6 months, which was in turn associated with a significant increased risk of subsequent exacerbation of PEs at 12 months in boys, whereas psychological distress at 12-month follow-up was significantly predicted by pre-existing PEs in girls.

The current results showed that the experience of PEs at baseline tended to temporally precede prospective increases in psychological distress (at 6 months), which had in turn led to further exacerbation of psychological distress at 12 months of follow-up. This supports previous findings that subclinical psychotic symptoms can provoke negative emotions and distress in non-help-seeking community-based young people [27, 28]. However, temporally primary psychological distress symptoms were not a significant predictor (Standardized Beta = 0.06, *p* = 0.125) for the development of later psychotic symptoms in the whole sample, but non-significant effect is plausible to have arisen from a lack of statistical power. Previous findings showed that, in nonclinical adults, stress has a significant effect on the emergence of PEs [76], and dysphoria (a combination of depression and anxiety) is considered a potential contributor to the occurrence of positive psychotic symptoms [77–79]. Concurrent with the present findings, a prospective cohort study in a large community sample of adolescents and young adults with a 6-year follow-up demonstrated that PEs were linked to a significant increase in depressive symptoms at 18 years, but no link was shown between depressive symptoms at 12 years and PEs at 18 years, thus suggesting that depression in youth do not seem to enhance subsequent risk of PEs [73]. It is of note, however, that the moderating effect of sex was not explored in this study. Another study using cross-lagged path modeling was not able to confirm the hypothesized longitudinal bidirectional association between subclinical psychotic symptoms and depression in a clinical sample of adolescents with major depression [32].

Another relevant finding of the current study is that temporal patterns between adolescent distress and psychotic symptoms differed for girls and boys. Adolescent girls who initially presented with PEs had an elevated risk of developing later psychological distress (i.e., depression, anxiety and stress), and adolescent boys who firstly endorsed psychological distress were at higher risk of experiencing new onset psychotic symptoms over time. Yet, little is reported in the literature about whether any sex differences in the longitudinal associations between PEs and distress could prove to exist and be informative for prevention and intervention efforts. There is some evidence that females with psychotic disorders present with worse depressive symptoms [80] and have increased vulnerability to stress levels [81] than males. Inconsistent with our results, a recent study by Stainton et al. [82] demonstrated that depressive, anxiety, and stress symptoms contributed equally and significantly to the prediction of PEs in both male and female adolescents from the general population. In the present sample, PEs preceded subsequent increases in distress during follow-up in both boys and girls; whereas distress was a significant predictor of later PEs only in boys. Previous findings pointed to the complex nature of sex disparities in adolescent mental health, which remains largely poorly understood [53]. The sex-based differences observed in the prospective relationship between distress and PEs can be partly explained by a variety of socioenvironmental and cultural factors. A large national survey among Tunisian high school adolescents aged 16 to 18 years showed that boys reported significantly greater lifetime prevalence of cannabis use than girls (16.1% versus 2.5%) [83]. This might explain our findings, as cannabis is consistently shown to be a strong predictor of psychosis onset in vulnerable individuals [84]. In addition, male adolescents have a significantly higher likelihood of being victims of child abuse in Tunisia [85], which can in turn lead to increased proneness to develop psychotic symptoms in those who are more distressed [67, 86]. Finally, Tunisian males had poorer knowledge about mental illness and were less likely to seek help from professional sources than females, which can be explained by social norms and sex-based stereotypes, such as autonomy and strength, that are associated with negative attitudes toward help-seeking behaviors [87]. Failure to seek help or disclose distress can contribute to negative mental health consequences, including the onset of psychotic symptoms. Overall, the mixed findings call for further investigations of the sex moderation effect on relationships between psychological distress and psychosis in different populations and settings.

### Study limitations

Our findings should be considered in light of a number of limitations. Data were gathered using convenience sampling and self-report measures, which may affect the representativeness of the sample and lead to response bias. Only community adolescents aged 13–18 years and from a single low-to-middle income Arab country (i.e. Tunisia) have been involved in the study, which may limit the generalization of findings to help-seeking and clinical adolescents, emerging adults, as well as those from other countries and cultures (e.g., high-income countries [88], non-Arab countries [89]). Further studies should seek to replicate the findings in these different samples. Certain potential confounding factors were not accounted for in this study’s analyses, such as genetic load, negative symptoms, cognitive functioning, traumatic experiences, or substance use [1, 67, 90, 91]. Given the sample is skewed towards girls (61%), it may be that boys with greater distress / PEs were more likely to refuse or drop out. Finally, analysis using RI-CLPM methodology was not possible with the present dataset; therefore, between-person confounding could not be controlled for.

### Clinical and research implications

This study builds on and expands previous research efforts by showing that adolescents who experience PEs are at a heightened risk for distressing outcomes in the form of depression, anxiety, and stress. Besides, distress seems to be, in itself, a worsening factor for PEs, contributing to their persistence and/or deterioration over time. These patterns of associations suggest that one factor could be an early marker for the other, and warrant routine screening for one outcome when the other is present, specifically in adolescent populations. Interestingly, the two factors were found to predict each other differently across sex, with persistent psychological distress at 6 months temporally preceding an exacerbation of PEs at 12 months in boys, and PEs at 6 months being at the origin of later psychological distress in girls. These findings suggest that clinicians and support workers are recommended to take into account different social risk profiles for boys and girls when considering interventions to address PEs and distress in adolescents. As for research perspectives, long-term follow-up studies (e.g., birth cohorts) still need to study the different possible pathways by which the two constructs could influence each other over longer periods of time. It is also crucial to investigate and better understand factors other than sex, such as a wider age range (adolescence versus early adulthood), that may moderate the relationships between psychosis and distress. Finally, the field may benefit from future experimental research testing whether targeting one of the two conditions (i.e., PEs and psychological distress) could reduce the incidence of the other in adolescents.

## Conclusion

Using advanced methodological approaches, this study contributes to the body of knowledge on this topic by (1) exploring possible bidirectional influences of PEs and distress, and (2) examining potential moderating effects of sex in these relationships. Beyond there general significance, these findings also enhance our understanding of these dynamics within cultures that have been significantly underexplored, thereby contributing to the corpus of cross-cultural research in psychiatry. Results showed that the predictive associations between variables varied across sex. Baseline PEs were found to drive psychological distress at 6 months, which in turn has led to a further increase in PEs at 12 months among adolescent girls. The reverse path leading from reported psychological distress at 6 months to later aggravation of PEs was significant among adolescent boys. These findings have important implications for the understanding of temporal relationships between PEs and psychopathology in adolescence, and for effectively guiding the timing and selection of preventive and interventive strategies.

## Data Availability

The datasets generated and/or analyzed during the current study are not publicly available due to restrictions from the ethics committee but are available from the corresponding author on reasonable request.

## References

[1] Linscott R, Van Os J. An updated and conservative systematic review and meta-analysis of epidemiological evidence on psychotic experiences in children and adults: on the pathway from proneness to persistence to dimensional expression across mental disorders. Psychol Med. 2013;43(6):1133–49. 22850401 10.1017/S0033291712001626

[2] McGrath JJ, Saha S, Al-Hamzawi A, Alonso J, Bromet EJ, Bruffaerts R, Caldas-de-Almeida JM, Chiu WT, de Jonge P, Fayyad J. Psychotic experiences in the general population: a cross-national analysis based on 31 261 respondents from 18 countries. JAMA Psychiatry. 2015;72(7):697–705. 26018466 10.1001/jamapsychiatry.2015.0575 PMC5120396

[3] Kelleher I, Connor D, Clarke MC, Devlin N, Harley M, Cannon M. Prevalence of psychotic symptoms in childhood and adolescence: a systematic review and meta-analysis of population-based studies. Psychol Med. 2012;42(9):1857–63. 22225730 10.1017/S0033291711002960

[4] Van Os J, Linscott RJ, Myin-Germeys I, Delespaul P, Krabbendam L. A systematic review and meta-analysis of the psychosis continuum: evidence for a psychosis proneness–persistence–impairment model of psychotic disorder. Psychol Med. 2009;39(2):179–95. 18606047 10.1017/S0033291708003814

[5] Fekih-Romdhane F, Pandi-Perumal SR, Conus P, Krebs MO, Cheour M, Seeman MV, Jahrami HA. Prevalence and risk factors of self-reported psychotic experiences among high school and college students: a systematic review, meta-analysis, and meta-regression. Acta Psychiatr Scand. 2022;146(6):492–514. 36000793 10.1111/acps.13494

[6] Kaymaz N, Drukker M, Lieb R, Wittchen H-U, Werbeloff N, Weiser M, Lataster T, Van Os J. Do subthreshold psychotic experiences predict clinical outcomes in unselected non-help-seeking population-based samples? A systematic review and meta-analysis, enriched with new results. Psychol Med. 2012;42(11):2239–53. 22260930 10.1017/S0033291711002911

[7] Veijola J, Mäki P, Jääskeläinen E, Koivukangas J, Moilanen I, Taanila A, Nordström T, Hurtig T, Kiviniemi V, Mukkala S. Young people at risk for psychosis: case finding and sample characteristics of the Oulu brain and Mind Study. Early Interv Psychiat. 2013;7(2):146–54. 10.1111/j.1751-7893.2012.00360.x 22672385

[8] Kelleher I, Cannon M. A neural efficiency-threshold model to understand psychotic experiences. Psychol Med. 2021;51(11):1777–82. 34078493 10.1017/S0033291721001495

[9] Kelleher I, Murtagh A, Molloy C, Roddy S, Clarke MC, Harley M, Cannon M. Identification and characterization of prodromal risk syndromes in young adolescents in the community: a population-based clinical interview study. Schizophr Bull. 2012;38(2):239–46. 22101962 10.1093/schbul/sbr164 PMC3283157

[10] Scott J, Martin G, Bor W, Sawyer M, Clark J, McGrath J. The prevalence and correlates of hallucinations in Australian adolescents: results from a national survey. Schizophr Res. 2009;107(2–3):179–85. 19046858 10.1016/j.schres.2008.11.002

[11] Laurens K, West S, Murray R, Hodgins S. Psychotic-like experiences and other antecedents of schizophrenia in children aged 9–12 years: a comparison of ethnic and migrant groups in the United Kingdom. Psychol Med. 2008;38(8):1103–11. 17935641 10.1017/S0033291707001845

[12] Rubio JM, Sanjuán J, Flórez-Salamanca L, Cuesta MJ. Examining the course of hallucinatory experiences in children and adolescents: a systematic review. Schizophr Res. 2012;138(2–3):248–54. 22464200 10.1016/j.schres.2012.03.012

[13] Bhavsar V, McGuire P, MacCabe J, Oliver D, Fusar-Poli P. A systematic review and meta‐analysis of mental health service use in people who report psychotic experiences. Early Interv Psychiat. 2018;12(3):275–85. 10.1111/eip.12464 PMC6001621 28805304

[14] Oh H, Waldman K, Stubbs B, Koyanagi A. Psychotic experiences in the context of mood and anxiety disorders and their associations with health outcomes among people of color in the United States. J Psychosom Res. 2019;118:27–33. 30782351 10.1016/j.jpsychores.2019.01.004

[15] DeVylder J, Burnette D, Yang L. Co-occurrence of psychotic experiences and common mental health conditions across four racially and ethnically diverse population samples. Psychol Med. 2014;44(16):3503–13. 25065632 10.1017/S0033291714000944

[16] Fekih-Romdhane F, Malaeb D, Loch AA, Farah N, Stambouli M, Cheour M, Obeid S, Hallit S. Problematic smartphone use mediates the pathway from suicidal ideation to positive psychotic experiences: a large cross-sectional, population-based study. Int J Mental Health Addict. 2023; 2023:1–18. 10.1007/s11469-023-01028-8 PMC9930705 36820017

[17] Fekih-Romdhane F, Sassi H, Cheour M. The relationship between social media addiction and psychotic-like experiences in a large nonclinical student sample. Psychosis. 2021;13(4):349–60.

[18] Fekih-Romdhane F, Jahrami H, Away R, Trabelsi K, Pandi-Perumal SR, Seeman MV, Hallit S, Cheour M. The relationship between technology addictions and schizotypal traits: mediating roles of depression, anxiety, and stress. BMC Psychiatry. 2023;23(1):1–9. 36698079 10.1186/s12888-023-04563-9 PMC9875437

[19] Fekih-Romdhane F, Lamloum E, Loch AA, Cherif W, Cheour M, Hallit S. The relationship between internet gaming disorder and psychotic experiences: cyberbullying and insomnia severity as mediators. BMC Psychiatry. 2023;23(1):857. 37978468 10.1186/s12888-023-05363-x PMC10657007

[20] Fekih-Romdhane F. Moderating effect of alexithymia between problem gambling and psychotic experiences in university students. BMC Psychiatry 2024. 10.1186/s12888-023-05472-7 PMC10765704 38172817

[21] Farah N, Obeid S, Malaeb D, Haddad C, Fekih-Romdhane F, Hallit S. Mediation effect of insomnia symptoms between positive psychotic like experiences and suicidal ideation among Lebanese young adults. BMC Psychiatry. 2023;23(1):1–10. 37081441 10.1186/s12888-023-04778-w PMC10116113

[22] Fekih-Romdhane F, Jahrami H, Alhuwailah A, Fawaz M, Shuwiekh HAM, Helmy M, Mohammed Hassan IH, Naser AY, Zarrouq B, Chebly M et al. Cross-country validation of the Arabic Version of the Prodromal questionnaire–brief (PQ-B) in young adults from the General Population of the Middle East and North Africa (MENA) Region. Int J Mental Health Addict 2023.

[23] Sharifi V, Eaton WW, Wu LT, Roth KB, Burchett BM, Mojtabai R. Psychotic experiences and risk of death in the general population: 24–27 year follow-up of the Epidemiologic Catchment Area study. Br J Psychiatry. 2015;207(1):30–6. 25953893 10.1192/bjp.bp.113.143198 PMC4486819

[24] Navarro-Mateu F, Alonso J, Lim CC, Saha S, Aguilar‐Gaxiola S, Al‐Hamzawi A, Andrade LH, Bromet EJ, Bruffaerts R, Chatterji S. The association between psychotic experiences and disability: results from the WHO World Mental Health surveys. Acta Psychiatrica Scandinavica. 2017;136(1):74–84. 28542726 10.1111/acps.12749 PMC5664954

[25] Martin G, Thomas H, Andrews T, Hasking P, Scott JG. Psychotic experiences and psychological distress predict contemporaneous and future non-suicidal self-injury and suicide attempts in a sample of Australian school-based adolescents. Psychol Med. 2015;45(2):429–37. 25065410 10.1017/S0033291714001615

[26] Mirowsky J, Ross CE. Social causes of psychological distress. In: 2nd ed edn. Hawthorne, N.Y.: Aldine de Gruyter Hawthorne, N.Y.; 2003.

[27] Healy C, Brannigan R, Dooley N, Coughlan H, Clarke M, Kelleher I, Cannon M. Childhood and adolescent psychotic experiences and risk of mental disorder: a systematic review and meta-analysis. Psychol Med. 2019;49(10):1589–99. 31088578 10.1017/S0033291719000485

[28] Carey E, Gillan D, Healy C, Dooley N, Campbell D, McGrane J, O’Neill A, Coughlan H, Clarke M, Kelleher I. Early adult mental health, functional and neuropsychological outcomes of young people who have reported psychotic experiences: a 10-year longitudinal study. Psychol Med. 2021;51(11):1861–9. 32216843 10.1017/S0033291720000616

[29] Hielscher E, Connell M, Lawrence D, Zubrick SR, Hafekost J, Scott JG. Prevalence and correlates of psychotic experiences in a nationally representative sample of Australian adolescents. Australian New Z J Psychiatry. 2018;52(8):768–81. 29992826 10.1177/0004867418785036

[30] McGrath JJ, Saha S, Al-Hamzawi A, Andrade L, Benjet C, Bromet EJ, Browne MO, Caldas de Almeida JM, Chiu WT, Demyttenaere K. The bidirectional associations between psychotic experiences and DSM-IV mental disorders. Am J Psychiatry. 2016;173(10):997–1006. 26988628 10.1176/appi.ajp.2016.15101293 PMC5175400

[31] Stochl J, Khandaker G, Lewis G, Perez J, Goodyer I, Zammit S, Sullivan S, Croudace T, Jones P. Mood, anxiety and psychotic phenomena measure a common psychopathological factor. Psychol Med. 2015;45(7):1483–93. 25394403 10.1017/S003329171400261X

[32] Wigman J, Lin A, Vollebergh WA, van Os J, Raaijmakers QA, Nelson B, Baksheev G, Yung A. Subclinical psychosis and depression: co-occurring phenomena that do not predict each other over time. Schizophr Res. 2011;130(1–3):277–81. 21458235 10.1016/j.schres.2011.03.003

[33] Polanczyk G, Moffitt TE, Arseneault L, Cannon M, Ambler A, Keefe RS, Houts R, Odgers CL, Caspi A. Etiological and clinical features of childhood psychotic symptoms: results from a birth cohort. Arch Gen Psychiatry. 2010;67(4):328–38. 20368509 10.1001/archgenpsychiatry.2010.14 PMC3776482

[34] van Rossum I, Dominguez M, Wittchen H-U, van Os J. Affective dysregulation and reality distortion: a 10-year prospective study of their association and clinical relevance. Schizophr Bull. 2011;37(3):561–71. 19793794 10.1093/schbul/sbp101 PMC3080695

[35] Dhossche D, Ferdinand R, van der Ende J, Hofstra M, Verhulst F. Diagnostic outcome of self-reported hallucinations in a community sample of adolescents. Psychol Med. 2002;32(4):619–27. 12102376 10.1017/s003329170200555x

[36] Scott J, Martin G, Welham J, Bor W, Najman J, O’Callaghan M, Williams G, Aird R, McGrath J. Psychopathology during childhood and adolescence predicts delusional-like experiences in adults: a 21-year birth cohort study. Am J Psychiatry. 2009;166(5):567–74. 19339357 10.1176/appi.ajp.2008.08081182

[37] Cannon M, Caspi A, Moffitt TE, Harrington H, Taylor A, Murray RM, Poulton R. Evidence for early-childhood, pan-developmental impairment specific to schizophreniform disorder: results from a longitudinal birth cohort. Arch Gen Psychiatry. 2002;59(5):449–56. 11982449 10.1001/archpsyc.59.5.449

[38] Wiles NJ, Zammit S, Bebbington P, Singleton N, Meltzer H, Lewis G. Self-reported psychotic symptoms in the general population: results from the longitudinal study of the British National Psychiatric Morbidity Survey. Br J Psychiatry. 2006;188(6):519–26. 16738341 10.1192/bjp.bp.105.012179

[39] Weiser M, Lubin G, Caspi A, Rabinowitz J, Shmushkevitz M, Yoffe R, Werbeloff N, Halperin D, Davidson M. Dysthymia in male adolescents is associated with increased risk of later hospitalization for psychotic disorders: a historical-prospective cohort study. Early Interv Psychiat. 2008;2(2):67–72. 10.1111/j.1751-7893.2008.00060.x 21352135

[40] Kessler RC, Avenevoli S, Merikangas KR. Mood disorders in children and adolescents: an epidemiologic perspective. Biol Psychiatry. 2001;49(12):1002–14. 11430842 10.1016/s0006-3223(01)01129-5

[41] Hyde JS, Mezulis AH. Gender differences in depression: biological, affective, cognitive, and sociocultural factors. Harv Rev Psychiatry. 2020;28(1):4–13. 31913978 10.1097/HRP.0000000000000230

[42] Barajas A, Ochoa S, Obiols JE, Lalucat-Jo L. Gender differences in individuals at high-risk of psychosis: a comprehensive literature review. Sci World J 2015;2015:430735. 10.1155/2015/430735 PMC4312997 25685840

[43] Van Os J, Hanssen M, Bijl RV, Ravelli A. Strauss (1969) revisited: a psychosis continuum in the general population? Schizophr Res. 2000;45(1–2):11–20. 10978868 10.1016/s0920-9964(99)00224-8

[44] Laurens KR, Hodgins S, Maughan B, Murray RM, Rutter ML, Taylor EA. Community screening for psychotic-like experiences and other putative antecedents of schizophrenia in children aged 9–12 years. Schizophr Res. 2007;90(1–3):130–46. 17207968 10.1016/j.schres.2006.11.006

[45] Poulton R, Caspi A, Moffitt TE, Cannon M, Murray R, Harrington H. Children’s self-reported psychotic symptoms and adult schizophreniform disorder: a 15-year longitudinal study. Arch Gen Psychiatry. 2000;57(11):1053–8. 11074871 10.1001/archpsyc.57.11.1053

[46] Ronald A, Sieradzka D, Cardno AG, Haworth CM, McGuire P, Freeman D. Characterization of psychotic experiences in adolescence using the specific psychotic experiences questionnaire: findings from a study of 5000 16-year-old twins. Schizophr Bull. 2014;40(4):868–77. 24062593 10.1093/schbul/sbt106 PMC4059437

[47] Scott J, Welham J, Martin G, Bor W, Najman J, O’Callaghan M, Williams G, Aird R, Mcgrath J. Demographic correlates of psychotic-like experiences in young Australian adults. Acta Psychiatr Scand. 2008;118(3):230–7. 18518864 10.1111/j.1600-0447.2008.01214.x

[48] Solmi M, Radua J, Olivola M, Croce E, Soardo L, Salazar de Pablo G, Il Shin J, Kirkbride JB, Jones P, Kim JH. Age at onset of mental disorders worldwide: large-scale meta-analysis of 192 epidemiological studies. Mol Psychiatry. 2022;27(1):281–95. 34079068 10.1038/s41380-021-01161-7 PMC8960395

[49] Koyanagi A, Oh H, Stickley A, Haro J, DeVylder J. Risk and functional significance of psychotic experiences among individuals with depression in 44 low-and middle-income countries. Psychol Med. 2016;46(12):2655–65. 27377628 10.1017/S0033291716001422

[50] Bhavsar V, Dorrington S, Morgan C, Hatch SL, McGuire P, Fusar-Poli P, Mills J, MacCabe JH, Hotopf M. Psychotic experiences, psychiatric comorbidity and mental health need in the general population: a cross-sectional and cohort study in Southeast London. Psychol Med. 2021;51(1):147–57. 31713511 10.1017/S0033291719003106 PMC7116680

[51] Jovanović V, Rudnev M, Iqbal N, Rice SPM, Żemojtel-Piotrowska M. Cross-cultural measurement of positive and negative emotions in Adolescence: evidence from three countries. J Happiness Stud. 2022;23(7):3143–60. 35645608 10.1007/s10902-022-00521-6 PMC9123922

[52] Alizadeh BZ, Bartels-Velthuis AA, Bruggeman R, Cahn W, Delespaul P, Fonseca-Pedrero E, Jaya ES, Kahn RS, Lincoln TM, Luykx JJ, et al. Comparing psychotic experiences in low-and-middle-income-countries and high-income-countries with a focus on measurement invariance. Psychol Med. 2022;52(8):1509–16. 33023691 10.1017/S0033291720003323

[53] Campbell OLK, Bann D, Patalay P. The gender gap in adolescent mental health: a cross-national investigation of 566,829 adolescents across 73 countries. SSM - Popul Health. 2021;13:100742. 33748389 10.1016/j.ssmph.2021.100742 PMC7960541

[54] Fekih-Romdhane F, Stambouli M, Malaeb D, Farah N, Cheour M, Obeid S, Hallit S. Insomnia and distress as mediators on the relationship from cyber-victimization to self-reported psychotic experiences: a binational study from Tunisia and Lebanon. BMC Psychiatry. 2023;23(1):524. 37475011 10.1186/s12888-023-05019-w PMC10360279

[55] Rahme C, El Kadri N, Haddad C, Fekih-Romdhane F, Obeid S, Hallit S. Exploring the association between lifetime traumatic experiences and positive psychotic symptoms in a group of long-stay patients with schizophrenia: the mediating effect of depression, anxiety, and distress. BMC Psychiatry. 2023;23(1):29. 36635691 10.1186/s12888-023-04531-3 PMC9835034

[56] Fekih-Romdhane F, Farah N, Malaeb D, Cheour M, Obeid S, Hallit S. Validation of the Arabic Version of the Community Assessment of psychic experiences (CAPE-42) in a large sample of young adults from the General Population. Int J Mental Health Addict 2023:1–18. 10.1007/s11469-023-01028-8 PMC9930705 36820017

[57] Fekih-Romdhane F, El Hadathy D, González-Nuevo C, Malaeb D, Barakat H, Hallit S. Development and preliminary validation of the Postpartum psychotic experiences Scale (PPES). Psychiatry Res. 2023;329:115543. 37839316 10.1016/j.psychres.2023.115543

[58] Fekih-Romdhane F, Jahrami H, Alhuwailah A, Fawaz M, Shuwiekh HAM, Helmy M, Mohammed Hassan IH, Naser AY, Zarrouq B, Chebly M. Cross-country validation of the Arabic Version of the Prodromal questionnaire–brief (PQ-B) in young adults from the general population of the Middle East and North Africa (MENA) region. Int J Mental Health Addict 2023:1–21.

[59] Fekih-Romdhane F, Houissa L, Cheour M, Hallit S, Loch AA. Body image as a mediator in the relationship between psychotic experiences and later disordered eating: a 12-month longitudinal study in high school adolescents. Int J Soc Psychiatry 2023:00207640231218686. 10.1177/00207640231218686 38160417

[60] Fekih-Romdhane F, Sassi H, Ennaifer S, Tira S, Cheour M. Prevalence and correlates of psychotic like experiences in a large community sample of young adults in Tunisia. Commun Ment Health J. 2020;56(6):991–1003. 10.1007/s10597-019-00542-1 31900754

[61] Fekih-Romdhane F, Hakiri A, Stambouli M, Cherif W, Away R, Amri A, Cheour M, Hallit S. Schizotypal traits in a large sample of high-school and university students from Tunisia: correlates and measurement invariance of the arabic schizotypal personality questionnaire across age and sex. BMC Psychiatry. 2023;23(1):447. 37340441 10.1186/s12888-023-04942-2 PMC10283320

[62] Fekih-Romdhane F, Saidi M, Chaabane MA, Cheour M. Knowledge, attitude and behaviours toward people with mental illness among Tunisian nursing students and nonhealth care students: a cross-sectional study. Collegian. 2022;29(4):500–9.

[63] Konings M, Bak M, Hanssen M, Van Os J, Krabbendam L. Validity and reliability of the CAPE: a self-report instrument for the measurement of psychotic experiences in the general population. Acta Psychiatr Scand. 2006;114(1):55–61. 16774662 10.1111/j.1600-0447.2005.00741.x

[64] Brenner K, Schmitz N, Pawliuk N, Fathalli F, Joober R, Ciampi A, King S. Validation of the English and French versions of the Community Assessment of psychic experiences (CAPE) with a Montreal community sample. Schizophr Res. 2007;95(1–3):86–95. 17693059 10.1016/j.schres.2007.06.017

[65] Fekih-Romdhane F, Nsibi T, Sassi H, Cheour M. Link between childhood trauma and psychotic-like experiences in non-affected siblings of schizophrenia patients: a case-control study. Early Interv Psychiatry. 2021;15(5):1154–66. 33034164 10.1111/eip.13054

[66] Fekih-Romdhane F, Sassi H, Ennaifer S, Tira S, Cheour M. Prevalence and correlates of psychotic like experiences in a large community sample of young adults in Tunisia. Community Ment Health J. 2020;56(6):991–1003. 31900754 10.1007/s10597-019-00542-1

[67] Fekih-Romdhane F, Tira S, Cheour M. Childhood sexual abuse as a potential predictor of psychotic like experiences in Tunisian college students. Psychiatry Res. 2019;275:181–8. 30925305 10.1016/j.psychres.2019.03.034

[68] Lovibond PF, Lovibond SH. The structure of negative emotional states: comparison of the Depression anxiety stress scales (DASS) with the Beck Depression and anxiety inventories. Behav Res Ther. 1995;33(3):335–43. 7726811 10.1016/0005-7967(94)00075-u

[69] Antony MM, Bieling PJ, Cox BJ, Enns MW, Swinson RP. Psychometric properties of the 42-item and 21-item versions of the depression anxiety stress scales in clinical groups and a community sample. Psychol Assess. 1998;10(2):176.

[70] Mellor D, Vinet EV, Xu X, Bt Mamat NH, Richardson B, Román F. Factorial invariance of the DASS-21 among adolescents in four countries. Eur J Psychol Assess. 2015;31(2):138–42.

[71] George D. SPSS for windows step by step: a simple study guide and reference, 17.0 update, 10/e. Pearson Education India; 2011.

[72] Orth U, Meier LL, Buhler JL, Dapp LC, Krauss S, Messerli D, Robins RW. Effect size guidelines for cross-lagged effects. Psychol Methods. 2024;29(2):421–33. 35737548 10.1037/met0000499

[73] Sullivan SA, Wiles N, Kounali D, Lewis G, Heron J, Cannon M, Mahedy L, Jones PB, Stochl J, Zammit S. Longitudinal associations between adolescent psychotic experiences and depressive symptoms. PLoS One. 2014;9(8):e105758. 25162230 10.1371/journal.pone.0105758 PMC4146535

[74] Kelleher I, Keeley H, Corcoran P, Lynch F, Fitzpatrick C, Devlin N, Molloy C, Roddy S, Clarke MC, Harley M. Clinicopathological significance of psychotic experiences in non-psychotic young people: evidence from four population-based studies. Br J Psychiatry. 2012;201(1):26–32. 22500011 10.1192/bjp.bp.111.101543

[75] Hanssen M, Peeters F, Krabbendam L, Radstake S, Verdoux H, Van Os J. How psychotic are individuals with non-psychotic disorders? Soc Psychiatry Psychiatr Epidemiol. 2003;38:149–54. 12616313 10.1007/s00127-003-0622-7

[76] Crowe S, Barot J, Caldow S, d’Aspromonte J, Dell’Orso J, Di Clemente A, Hanson K, Kellett M, Makhlota S, McIvor B. The effect of caffeine and stress on auditory hallucinations in a non-clinical sample. Pers Indiv Differ. 2011;50(5):626–30.

[77] Cella M, Cooper A, Dymond SO, Reed P. The relationship between dysphoria and proneness to hallucination and delusions among young adults. Compr Psychiatr. 2008;49(6):544–50. 10.1016/j.comppsych.2008.02.011 18970902

[78] Paulik G, Badcock JC, Maybery MT. The multifactorial structure of the predisposition to hallucinate and associations with anxiety, depression and stress. Pers Indiv Differ. 2006;41(6):1067–76.

[79] Guillem F, Pampoulova T, Stip E, Lalonde P, Todorov C. The relationships between symptom dimensions and dysphoria in schizophrenia. Schizophr Res. 2005;75(1):83–96. 15820327 10.1016/j.schres.2004.06.018

[80] Abel KM, Drake R, Goldstein JM. Sex differences in schizophrenia. Int Rev Psychiatry. 2010;22(5):417–28. 21047156 10.3109/09540261.2010.515205

[81] Myin-Germeys I, Krabbendam L, Delespaul P, Van Os J. Sex differences in emotional reactivity to daily life stress in psychosis. J Clin Psychiatry. 2004;65(6):805–9. 15291657 10.4088/jcp.v65n0611

[82] Stainton A, Chisholm K, Woodall T, Hallett D, Reniers RL, Lin A, Wood SJ. Gender differences in the experience of psychotic-like experiences and their associated factors: a study of adolescents from the general population. Schizophr Res. 2021;228:410–6. 33556674 10.1016/j.schres.2021.01.008

[83] Mallekh R, Rejaibi S, Silini A, Zid M, Slema IB, Zoghlami N, Youssef SB, Zribi M, Salah NB, Aounallah-Skhiri H. Cannabis use in Tunisian high school adolescents: MedSPAD 2021. Eur Psychiatry. 2023;66(S1):S530–530.

[84] Fekih-Romdhane F, Hakiri A, Fadhel SB, Cheour M. Cannabis use in subjects at Ultra high risk for psychosis. Presse Med. 2019;48(11 Pt 1):1229–36. 31732360 10.1016/j.lpm.2019.09.030

[85] Braham MY, Jedidi M, Hmila I, Masmoudi T, Souguir MK, Dhiab MB. Epidemiological aspects of child abuse and neglect in Sousse, Tunisia: a 10-year retrospective study. J Forensic Leg Med. 2018;54:121–6. 29413953 10.1016/j.jflm.2018.01.003

[86] Fekih-Romdhane F, Hakiri A, Cheour M. Childhood trauma in subjects at Ultra high risk for psychosis. Presse Med. 2019;48(3 Pt 1):243–9. 30711298 10.1016/j.lpm.2018.11.023

[87] Fekih-Romdhane F, Chebbi O, Sassi H, Cheour M. Knowledge, attitude and behaviours toward mental illness and help‐seeking in a large nonclinical Tunisian student sample. Early Interv Psychiat. 2021;15(5):1292–305. 10.1111/eip.13080 33300260

[88] McGrath JJ, Saha S, Al-Hamzawi A, Alonso J, Bromet EJ, Bruffaerts R, Caldas-de-Almeida JM, Chiu WT, de Jonge P, Fayyad J, et al. Psychotic experiences in the General Population: a cross-national analysis based on 31 261 respondents from 18 countries. JAMA Psychiatry. 2015;72(7):697–705. 26018466 10.1001/jamapsychiatry.2015.0575 PMC5120396

[89] Khaled SM, Brederoo SG, Yehya A, Alabdulla M, Woodruff PW, Sommer IE. Cross-cultural differences in Hallucinations: a comparison between Middle Eastern and European Community-based samples. Schizophr Bull. 2023;49(Supplement1):S13–24. 36840542 10.1093/schbul/sbac086 PMC9960011

[90] Addington J, Heinssen R. Prediction and prevention of psychosis in youth at clinical high risk. Ann Rev Clin Psychol. 2012;8:269–89. 22224837 10.1146/annurev-clinpsy-032511-143146

[91] Dickson H, Laurens KR, Cullen AE, Hodgins S. Meta-analyses of cognitive and motor function in youth aged 16 years and younger who subsequently develop schizophrenia. Psychol Med. 2012;42(4):743–55. 21896236 10.1017/S0033291711001693

